# Feelings of guilt among cancer patients and the usage of complementary or alternative medicine – A cross-sectional survey

**DOI:** 10.1017/S1478951524001718

**Published:** 2025-01-20

**Authors:** Sarah Sophie Meren, Lena Josfeld, Jozien Clazina Bahlmann, L. Fischer von Weikersthal, H. Männle, J. Huebner

**Affiliations:** 1 Hämatologie und Internistische Onkologie, Universitätsklinikum Jena Klinik für Innere Medizin II Friedrich-Schiller Universität Jena, Jena, Germany; 2 Nordwestdeutsches Tumorzentrum, Klinikum Oldenburg AöR, Oldenburg, Germany; 3 Praxis für Hämatologie und Internistische Onkologie, Gesundheitszentrum St. Marien GmbH, Amberg, Germany; 4 Gynäkologie und Geburtshilfe, Ortenau Klinikum Offenburg-Gengenbach Standort Offenburg Ebertplatz, Offenburg, Germany

**Keywords:** Guilt, neoplasm, complementary medicine, self-efficacy, patient activation

## Abstract

**Objectives:**

This study aimed to investigate the influence of feelings of guilt among cancer patients on their health behavior, with a specific focus on the use of complementary and alternative medicine (CAM).

**Methods:**

A multicentric cross-sectional study was conducted, involving 162 oncological patients, assessing sociodemographic variables, feelings of guilt, patient activation, self-efficacy, and CAM usage. The Shame-Guilt-Scale was employed to measure guilt, with subscales including punitive guilt, self-criticism (actions), moral perfectionism, and empathy-reparation. To assess patient activation and self-efficacy, we used the German Version of the Patient Activation Measure 13 and the Short Scale for Measuring General Safe-efficacy Beliefs, respectively. To evaluate CAM-usage, we used a standardized instrument from the working group Prevention and Integrative Oncology of the German Cancer Society. Statistical analyses, including regression models, were employed to examine potential associations.

**Results:**

Female gender was associated with more frequent CAM usage. Regarding holistic and mind-body-methods, younger patients more often used these methods. No significant association was found between feelings of guilt and CAM usage. Patients experienced guilt most strongly related to empathy and reparation for their own actions.

**Significance of results:**

Our results do not support the hypothesis of a direct link between guilt and CAM usage. Guilt may be an important aspect in psychological support for cancer patients, yet, with respect to counselling on CAM, it does not play an important part to understand patients’ motivations.

## Introduction

Complementary and alternative methods have a high popularity among cancer patients and may even be on the rise according to survey data (Bauer et al. 2018; Hübner et al. 2022; Huebner et al. 2014b), while disclosure to the treating physician is rather low (Schütze et al. 2016). The employment of such methods may be due to the patients wishing to explore all potential options, indicative of a coping mechanism, or indicative of unfulfilled preferences in their current course of treatment (Alsharif 2021).

Complementary and alternative medicine (CAM) often is defined as methods coming from traditional medicine and naturopathy with alternative medicine being any method which is used instead of a conventional treatment. In contrast, complementary medicine is used in addition. By this definition, 1 method may be alternative in 1 patient and complementary in another. In our work, we use the definition of complementary or alternative medicine also used in the German S3 guideline on complementary medicine for cancer patients (Leitlinienprogramm Onkologie (Deutsche Krebsgesellschaft, Deutsche Krebshilfe, AWMF). While alternative medicine is any use of non-evidence-based methods instead of or along with evidence-based treatment, complementary medicine comprises additional methods for which some evidence exists, based on which a benefit–risk assessment for the individual patient may be performed.

The German S3 guideline on CAM in cancer care (Leitlinienprogramm Onkologie (Deutsche Krebsgesellschaft, Deutsche Krebshilfe, AWMF) recommends asking all cancer patients about their interest in and usage of CAM in regular terms. Thus, patients’ needs should be explored, and the risks of side effects and interactions with CAM should be reduced. The main aim of patients looking for information on CAM is to strengthen themselves and/or the immune system, to do something for themselves and to fight cancer directly or to detoxify. Less often, patients aim at reducing side effects of cancer treatment (Huebner et al. 2014a; Loquai et al. 2017).

To counsel patients in an optimal way, not only their aims but also motives to turn to CAM are important. Several studies, up to now including more than 4500 patients in different settings, assessed potential factors and motives in CAM usage. The type of CAM chosen does not depend on lay-etiological concepts patients have (Huebner et al. 2023). Moreover, we find significantly higher CAM usage in patients with high external locus of control but no correlation to internal locus of control (Ebel et al. 2015). Interest in CAM is associated with a higher self-efficacy and a higher patient activation. Yet, for actual CAM usage, this is not true (Ciarlo et al. 2021). In contrast, self-efficacy correlates with healthy nutrition and physical activity (Josfeld et al. 2022). Self-efficacy describes the estimate on one’s own competences, to act efficaciously in daily life, to cope with difficulties and barriers, and to overwhelm critical situations using one’s own forces (Bandura 1997; Hinz et al. 2006).

Patient activation involves 4 elements – knowledge, skills, confidence, and behaviors – that are critical for coping with chronic illness, reflecting the different levels of activation patients achieve in managing their own health (Hibbard and Tusler 2007).

Another concept discussed in the context of patients’ decision-making with respect to medical issues, is guilt.

Previous studies have focused on feelings of guilt in the context of chronic diseases, revealing a strong correlation between these factors (Cerna et al. 2022). Feelings of guilt are, among others, commonly observed emotions in cancer patients (Arian et al. 2021) and guilt belongs to the most often expressed emotions of cancer patients in social media (Park et al. 2020). Guilt, in medical context, refers to the retrospective negative evaluation of a former health related decision or action. It comprises different subscales as namely self-criticism (in regard to this decision or actions), moral perfectionism, empathy-reparation, and punitive guilt.

Patients with cancers associated with an unhealthy lifestyle might feel guilty. One commonly discussed example is the feeling of guilt in patients and former smokers with lung cancer (LoConte et al. 2008; Perloff et al. 2019; Shin et al. 2014; Siwik et al. 2022). Yet, our own work has shown that even in a group of patients with high risk behaviors (smoking and alcohol) only a minor part believes in this being the cause of their disease (Paul et al. 2013). In contrast, there is a rising number of patients believing in unhealthy nutrition and environmental toxins to be the cause (Ebel et al. 2015). Moreover, a growing number of cancer patients are turning to vegetarian and vegan diets, believing that their previous nutritional habits were not optimal. Similarly, Robertson et al. (2018) describe this as a feeling of guilt among cancer survivors for not achieving recommended levels of physical exercise.

Several studies have focused on survivor guilt among cancer patients, identified in those with ovarian and lung cancer (Glaser et al. 2019; Tetteh 2022). Subsequent studies have gone even further and examined the effect of guilt on health behavior, particularly moderate-to-vigorous intensity physical activity (MVPA), of breast cancer survivors over time, revealing that experiencing body-related guilt is associated with an immediate increase in MVPA (Castonguay et al. 2017).

In another context, Arian et al. (2024) describe existential guilt in cancer patients as “a deep and multidimensional concept that is correlated with concepts, such as in-/authenticity, existential anxiety, decisiveness, and personal and social responsibility.” A qualitative analysis of interviews reveals 3 main concepts (incompleteness, passivity, feelings of harm to self and others).

To our knowledge, so far, no studies from Western countries have been published assessing whether feeling guilt has any influence on CAM usage and type of CAM usage in cancer patients.

By investigating whether guilt influences CAM utilization, we may better understand patients’ emotional needs and preferences. Moreover, identifying a potential interplay between guilt and CAM usage may pave the way for the development of tailored interventions that address guilt-related needs of cancer patients that so far are not adequately addressed in cancer centers, thus empowering patients to make informed decisions that increase their well-being and improve their quality of life throughout their cancer journey.

## Methods

### Study design

This study is a prospective multicentric cross-sectional survey, which was carried out across 4 oncological centers in Germany. These centers are part of a larger group of centers cooperating regularly with our working group to conduct surveys on CAM usage. The acquisition of data for this study was conducted over the span of February 2023 until June 2023.

### Participants

Inclusion criteria were a diagnosis of cancer and sufficient proficiency in the German language to ensure that patients were capable of adequately understanding and responding to the questionnaire. Patients aged <18 years were excluded from this study. All patients visiting the day clinics of these centers in the respective time period were handed out the questionnaire. Participation in the survey was voluntary and anonymized. All participants provided informed consent by completing the questionnaire.

The questionnaire was returned in a closed box by the participants.

### Questionnaire

The questionnaire was composed of 5 parts (see eSupplement 1 for the German original version).

1. *Demographics.* Demographic data including gender, age, marital status, religion, education, time since initial cancer diagnosis as well as the type of cancer and previously received treatment. All questions were closed questions including a list of most frequent types of cancer and treatments including a free line to add other types or treatments.
2. *Patient activation.* The German Version of the Patient Activation Measure 13, or PAM13-D, is a reliable (*α* = .84) and validated translation of the short, 13-item form of the Patient Activation Measure, or “PAM” (Brenk-Franz et al. 2013; Djalali and Steurer-Stey 2014), which reflects a tool used to assess patient activation. It refers to the degree of knowledge, skills, and confidence in managing one’s health (Hibbard et al. 2004) and consists of 13 statements rated on a 4-point Likert scale (this statement is false, … is mostly false, … is rather correct, … is exactly correct) (Hibbard et al. 2005).
3. *Propensity to guilt.* To assess guilt proneness, we selectively considered 4 guilt-related subscales of the German version of the original Italian “Scala di Colpa e Vergogna” (Shame-Guilt-Scale (Battacchi et al. 2001; Suslow et al. 1999)). The original questionnaire consisted of 11 subscales with a total of 38 items. However, only 4 of those subscales (with a total of 12 items), namely self-criticism (in regard to actions), moral perfectionism, empathy-reparation and punitive guilt, are primarily intended to assess guilt-related personality traits and were therefore chosen selectively. To determine internal consistency, Suslow et al. (1999) performed a reliability estimate using the Cronbach’s alpha coefficient for each subscale. The guilt-related subscales revealed moderate to good reliability findings ranging from *α* = .5 to *α* = .72. The 12 statements were rated on a 5-point Likert scale ranging from “does not apply to me at all” to “applies to me completely.”
4. *Self-efficacy*. By using the “Allgemeine Selbstwirksamkeit Kurzskala” (Short Scale for Measuring General Safe-efficacy Beliefs), or “ASKU,” a 3-item tool for self-evaluation, employed in examining an individual’s subjective perception of their competency in addressing life challenges, was employed (Beierlein et al. 2012). The statements were rated on a 5-point Likert scale (ranging from “this is not the case at all” to “this is exactly the case”) and displayed a sufficient validity and reliability, the latter measured in 3 samples with the McDonald Omega coefficient ranging from *ω* = .81 to *ω* = .86.
5. *CAM.* CAM usage was assessed by the questionnaire recommended for regular assessment in all cancer patients in the German S3 guideline on complementary oncology (Leitlinienprogramm Onkologie (Deutsche Krebsgesellschaft, Deutsche Krebshilfe, AWMF)) which is based on a standardized instrument developed by the working group Prevention and Integrative Oncology of the German Cancer Society (Huebner et al. 2014b). Patients were presented with 15 different CAM methods (different types of micronutrients including vitamins and selenium, secondary plant extracts, herbs (with several lines to fill in the names of herbs used), traditional Chinese herbs, acupuncture/acupressure, ketogenic diet or fasting, Yoga, Tai chi and QiGong, mind-body-methods such as meditation or homeopathy). The patients were asked to mark all those methods they used currently or previously. A single cross option was provided for non-users.

### Statistics

Statistical analyses were performed using the program IBM SPSS Statistics Version 29.

To determine the overall PAM score, a sum score was generated, considering missing responses. The score was then further transformed, which enabled the classification of patients into 4 levels of activation, where levels 1 and 2 indicated a low level of activation and levels 3 and 4 indicated higher levels of patient activation. Self-efficacy was quantified by a mean value ranging from 1 to 5. The subscales of the Shame-Guilt-Scale were analyzed separately. For each subscale, sum scores were calculated. Missing data were addressed through imputation. Prior to analysis, variables were assessed for normal distribution.

Finally, we conducted a logistic regression analysis with “CAM usage” as the dependent variable. The independent variables were entered in 2 blocks: Block 1 included gender, age, and education, while Block 2 comprised level of activation, self-efficacy, and the subscales of the Guilt Scale (punitive guilt, self-criticism, and moral perfectionism). We repeated this analysis separately for biologically-based CAM and holistic and mind-body methods.

## Results

### Demographics

A total of 162 oncological patients participated in the study. The mean age of all participants was 60.8 years, ranging from 20 to 91 years. The majority of participants were female. A more detailed overview of the demographic characteristics of the study sample can be found in Table 1.

**Table 1.** Demographic data (*N* = 162)

Data		*N*	(%)
Gender	Female	97	59.9
	Male	65	40.1
Age	<50	30	21.1
	50–59	22	15.5
	60–69	52	36.6
	70–79	28	19.8
	80+	10	7
Marital status	Married	112	70
	Single	17	10.6
	Widowed	16	10
	Divorced	15	9.4
Level of education	Secondary school qualification (9th grade)	65	42.8
	Secondary school qualification (10th grade)	48	31.6
	University entrance diploma	21	13.8
	University degree	18	11.8
Religion	Catholic	73	46.8
	Protestant	52	33.3
	Atheist	27	17.3
	Orthodox	2	1.3
	Other	2	1.3
Type of cancer	Breast	38	24.5
	Leukemia, lymphoma	37	23.9
	Colorectal	21	13.6
	Gastrointestinal	16	10.3
	Urinary tract	12	7.8
	Gynecological	11	7.1
	Lung	9	5.8
	Head and neck	7	4.5
	Prostate	1	.6
	Others	3	1.9
Time since diagnosis	Less than 1 year	60	48
	1–3 years	33	26.4
	3–6 years	12	9.6
	More than 6 years	20	16
Previous treatment	Operation	92	58.6
	Chemotherapy	110	70.1
	Radiation therapy	47	29.9
	Hormonal therapy	18	11.5
	Immunotherapy or targeted therapy	39	24.8
	Others	5	3.2

Differences in *N* are due to missing information given by the patients.

Breast cancer patients were significantly correlated with higher use of biological-based (*r* = .453, *p* < .01) and holistic and mind-body (*r* = .508, *p* < .01) CAM methods.

### Guilt

The Shame-Guilt-Scale was completed by 157 (96.91%) patients in its entirety, who were included in further evaluation.

The 4 subscales of the Shame-Guilt-Scale are considered separately. Higher mean values are indicative of a higher proneness to guilt, whereas lower scores are indicative of a lower proneness to guilt. A detailed overview of the descriptive characteristics of the subscales can be found in Table 2.

**Table 2.** Mean, median, std. deviation, variance of feelings of guilt (*N* = 157)

	Punitive guilt	Self-criticism (actions)	Moral perfectionism	Empathy-reparation
N*	157	156	155	156
Mean	3.303	5.362	6.932	7.603
Median	3.0	6.0	7.0	8.0
Standard deviation	2.8340	3.6587	2.9127	3.2137
Variance	8.031	13.386	8.484	10.328

* One patient did not fill in part 2 and 3 of the scale.

### Patient activation and self-efficacy

*Patient activation.* The mean PAM13-D score (on a calibrated scale with a theoretical range from 0 to 100) was 70.10 (*SD* = 15.89). Of all the participants, 18.4% (*N* = 29) were categorized as Level 1 (6.4%) or Level 2 (12.0%), whereas 81.6% (*N* = 128) were categorized as Level 3 (33.8%) or Level 4 (47.8%).

*Self-efficacy.* The Short Scale for Measuring General Self-Efficacy Beliefs yielded a mean score of 3.86 (*N* = 156, *SD* = .89), ranging from a minimum of 1.0 point to a maximum of 5.0 points.

### Usage of CAM

**Figure 1.**
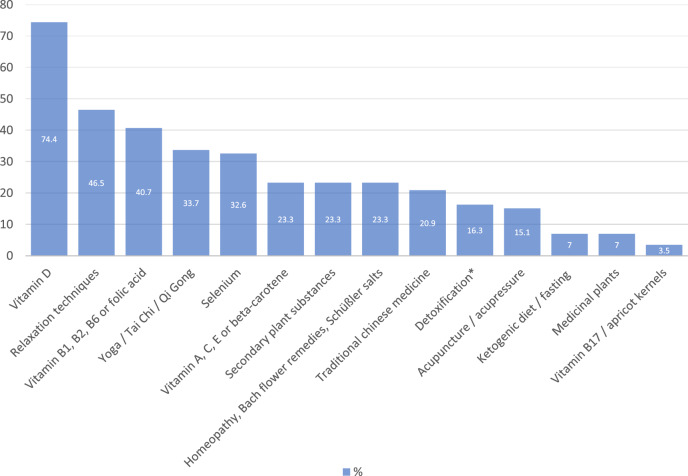
Types of CAM utilized among CAM-users (N = 86).

A total of 71 patients (45.2%) declared that they did not use any type of CAM. Eighty-six patients (54.8%) used at least 1 method, with 22 (14.0%) reporting using 1 method, 14 (8.9%) 2 methods, and 15 (9.6%) 3 methods. Five patients (3.1%) used 10 or more methods. The most popular CAM method chosen was vitamin D (74.4% of all CAM users), followed by relaxation methods (46.5%) and B-vitamins (40.70%) (Figure 1).

*Detoxification denotes methods to purge and remove alleged toxins from the body by inducing emesis, diarrhea taking (natural) substances or using enemas, or by activating circulation and metabolism.

The logistic regression analysis with “CAM usage” as the dependent variable included 2 blocks of independent variables. Block 1 comprised gender, age, and education, while block 2 included the level of activation, self-efficacy, and the subscales of the Guilt Scale (punitive guilt, self-criticism, and moral perfectionism). The logistic regression model assessing CAM usage as the dependent variable is significant (Chi square = 31.965, *p* < .001; *n* = 157), explaining 29.70% of the variance in CAM usage (Nagelkerke *R*^2^ = .297) and correctly classifying 72.2% of cases. The analysis revealed that male patients are significantly less likely to use CAM (*p* < .001). However, neither age nor education show a significant association with CAM use. Additionally, self-efficacy and patient activation levels do not significantly influence CAM usage. Similarly, none of the 3 subscales of guilt show a significant correlation with CAM usage (Table 3a).

The repetition of this analysis with biological-based CAM and holistic and mind-body methods did not reveal any major changes in the model (Tables 3b and 3c). Also considering biological-based and holistic plus mind-body methods, female patients significantly more often used both classes of CAM. One additional factor became significant in the model for holistic and mind-body-methods with younger patients significantly more often using these methods (*p* = .024).

**Table 3a.** Logistic regression analysis for demographic data, psychological data, guilt, and CAM usage (*N* = 157)

							95% CI for Exp(B)	
Variables in the equation	*B*	*SE*	Wald	*df*	Sig.	Exp(B)	Lower	Upper
*Gender*	−2.029	.446	20.714	1	<.001	.132	.055	.315
*Age*	−.012	.017	.503	1	.478	.988	.956	1.021
Higher Education*	.447	.538	.691	1	.406	1.563	.545	4.484
*Level Of Activation*			7.423	3	.060			
*ASKU_Mean*	−1.959	1.130	3.006	1	.083	.141	.015	1.291
*Subscale: Punitive Guilt*	−.325	1.041	.097	1	.755	.723	.094	5.559
*Subscale: Self-criticism*	.186	1.094	.029	1	.865	1.205	.141	10.288
*Subscale: Moral Perfectionism*	−.171	.292	.342	1	.559	.843	.476	1.494
*Constant*	.003	.068	.002	1	.965	1.003	.878	1.146

* Higher education: university entrance certificate or university degree.

**Table 3b.** Logistic regression analysis for demographic data, psychological data, guilt, and usage of biological-based CAM (*N* = 157)

							95% CI for Exp(B)	
Variables in the equation	*B*	*SE*	Wald	*df*	Sig.	Exp(B)	Lower	Upper
*Gender*	−1.690	.431	15.345	1	<.001	.184	.079	.430
*Age*	−.002	.016	.014	1	.904	.998	.968	1.030
Higher Education*	.360	.511	.496	1	.481	1.433	.527	3.899
*Level Of Activation*			5.367	3	.147			
*ASKU_Mean*	−1.831	1.100	2.770	1	.096	.160	.019	1.384
*Subscale: Punitive Guilt*	−.555	1.006	.304	1	.581	.574	.080	4.121
*Subscale: Self-criticism*	−.147	1.058	.019	1	.889	.863	.109	6.863
*Subscale: Moral Perfectionism*	−.185	.275	.452	1	.501	.831	.485	1.425
*Constant*	.048	.065	.559	1	.455	1.050	.925	1.191

* Higher education: university entrance certificate or university degree.

**Table 3c.** Logistic regression analysis for demographic data, psychological data, guilt, and usage of holistic and mind-body-methods (*N* = 157)

							95% CI for Exp(B)	
Variables in the equation	*B*	*SE*	Wald	*df*	Sig.	Exp(B)	Lower	Upper
*Gender*	−1.633	.436	14.051	1	<.001	.195	.083	.459
*Age*	−.039	.017	5.065	1	.024	.962	.929	.995
Higher Education*	.256	.555	.214	1	.644	1.292	.436	3.833
*Level Of Activation*			7.339	3	.062			
*ASKU_Mean*	−.413	1.016	.165	1	.684	.662	.090	4.843
*Subscale: Punitive Guilt*	.922	.938	.966	1	.326	2.515	.400	15.819
*Subscale: Self-criticism*	1.630	.994	2.689	1	.101	5.105	.727	35.831
*Subscale: Moral Perfectionism*	−.065	.274	.056	1	.813	.937	.548	1.602
*Constant*	.098	.069	1.996	1	.158	1.103	.963	1.263

* Higher education: university entrance certificate or university degree.

## Discussion

According to the literature available, this multicentric cross-sectional study is the initial attempt to evaluate correlations between feelings of guilt in cancer patients and their potential influence on the use of CAM. Contrary to our initial hypothesis, we found no significant association between feelings of guilt and CAM use.

However, we observed that female patients were more likely to use CAM and younger patients showed a preference for holistic and mind-body CAM methods.

No significant relationship was observed between the patient activation level, self-efficacy, and CAM usage.

More than half of our study population employed some form of CAM, which aligns with existing data on CAM use in oncological patients (Alsharif 2021; Ciarlo et al. 2021; Hübner et al. 2022).

In terms of demographic variables, our results indicate that female patients are more likely to use CAM. These findings are consistent with previous research highlighting gender differences in CAM utilization (Bauer et al. 2018; Huebner et al. 2023). In the past, studies have attributed these findings of women displaying higher user rates of CAM than men to a variety of factors, including general gender differences in the propensity to seek health care and higher health consciousness (Bertakis et al. 2000; Vaidya et al. 2012).

Furthermore, our analysis suggests that younger patients are more inclined to use holistic and mind-body CAM methods. These results align with the findings of Huebner et al. (2023), who reported similar trends in CAM preference among younger populations. The popularity of holistic and mind-body CAM methods has also been described in other recent studies (Matriz et al. 2024).

Another cross-sectional study conducted in Sweden reported similar demographic trends in CAM use among cancer patients (Wode et al. 2019).

While the results of the patient activation measure and self-efficacy are in concordance with previous studies in cancer patients (Hinz et al. 2019; Hübner et al. 2022; Huebner et al. 2014a; Kulpa et al. 2016; Lemanska et al. 2022; Welter et al. 2021), indicating a higher degree of proactiveness and engagement of patients in their own health care, our study found no significant correlation between CAM usage and levels of self-efficacy or patient activation.

This finding aligns with the notion that, while higher self-efficacy and patient activation are linked to an increased interest in CAM (Hübner et al. 2022), they do not necessarily translate into actual CAM usage (Ciarlo et al. 2021).

Finally, an anticipated link between guilt across all subscales and CAM usage was not observed. This finding diverges from earlier studies suggesting that emotions such as guilt significantly impact health behaviors and can play an essential role in health-related decision-making (Arian et al. 2021; Carpenter and Niedenthal 2018; Cerna et al. 2022; Zhang and Liao 2021). A recent study conducted by Tetteh (2022) on survivor guilt in ovarian cancer patients revealed a connection between guilt and certain health behaviors but not necessarily CAM usage.

While guilt is a prevalent emotion among patients with cancer (Arian et al. 2021; Park et al. 2020), it may not directly translate into the decision to use CAM. The absence of a significant connection between guilt and CAM usage in our study prompts us to focus on investigating the dynamics between other emotional or cognitive factors, patient decisions, and health-care approaches, which potentially play a more critical role in health behaviors.

The overall variance in CAM usage explained by our model additionally indicates that other unmeasured factors are likely to contribute to CAM use. Studies suggest that personal beliefs about the efficacy of CAM, social support, and level of communication with health-care providers about CAM are crucial determinants of CAM usage (Escudero-Vilaplana et al. 2023; Källman et al. 2023; Wode et al. 2019).

Understanding that guilt does not directly influence CAM usage can help clinicians focus on other psychological factors when advising patients with cancer on CAM. Interventions can be tailored to address specific emotional needs without assuming a direct link with CAM preferences.

For instance, psychological support could prioritize managing feelings of guilt in general rather than focusing on how these feelings might influence the patient’s decision on CAM usage.

Health-care providers should incorporate a more holistic approach to provide better care to patients.

### Limitations

The main limitation of our study is the relatively small sample size due to a low rate of participation by the patients addressed. Consequently, all data put forth shall rather be regarded as preliminary with further need of investigation. Additionally, the setting and timing in which the patients were participating in the study may be another limiting factor as the questionnaire was distributed before or after counselling sessions on cancer treatment which may influence the evaluation of some items of the questionnaire. This may be particularly evident when the patient has just received discouraging news.

Additionally, we did not conduct a power calculation prior to recruitment due to the initial hesitancy from our network of recruiting centers to participate in the survey. They expressed concerns about potential participant discomfort and embarrassment. Consequently, those willing to participate requested a relatively short recruitment period.

## Conclusion

To our knowledge, this study represents the primary endeavor to assess the association between feelings of guilt in patients with cancer and their use of CAM.

Although our findings revealed no direct association between guilt and CAM use, they shed light on the significant role of demographic variables, such as gender and age, in influencing CAM usage.

Moreover, this study explained only a fraction of the variance in CAM usage, suggesting that other unmeasured factors are likely to contribute to its use. Additionally, feelings of guilt have received relatively little attention in the context of cancer care, especially with regard to patients’ resulting health behaviors.

Therefore, our findings open the door to further exploration of potential influencing factors on CAM use as well as studying the influence of guilt on other health behavior-associated aspects.

Ultimately, this study contributes to a deeper understanding of the emotional experiences of patients with cancer, emphasizing the need for health-care providers to approach care with a sensitivity, adaptability, and patient-centered focus, as well as the importance of tailoring health-care interventions to individual patient profiles.

## Supporting information

Meren et al. supplementary material Meren et al. supplementary material

## Supplementary material

The supplementary material for this article can be found at https://doi.org/.10.1017/S1478951524001718.
