# Supplementary material for: Feelings of guilt among cancer patients and the usage of complementary or alternative medicine – A cross-sectional survey
Source: Palliat Support Care. 2025 Jan 20;23:e21. doi: 10.1017/S1478951524001718 (PMC13168776; doi:10.1017/S1478951524001718)
Supplement: Meren et al. supplementary material [file S1478951524001718sup001.docx]

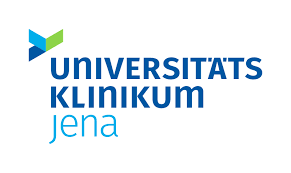


Datum: _____._____.________

Sehr geehrte*r Patient*in,

das Wissen über Krebserkrankungen ist heutzutage sehr umfangreich, dennoch gibt es nur wenige Studien, die den persönlichen Umgang mit jenen Erkrankungen untersuchen.

So eine schwerwiegende Erkrankung kann häufig die Psyche, Alltagstätigkeiten oder zwischenmenschlichen Beziehung von Betroffenen belasten, weshalb es von wachsender Wichtigkeit ist, Studien zunehmend in diese Richtung zu konzentrieren.

Mit dem folgenden Fragebogen möchten wir den Zusammenhang zwischen der Erkrankung und dem persönlichen Umgang mit jener auf verschiedenen Gefühlsebenen untersuchen und das Gefühlserleben des Patienten bestmöglich erfassen.

Aus diesem Grund werden Sie sowohl Fragen zu verschiedenen Therapiealternativen als auch Fragen mit eher psychologischem Hintergrund in diesem Fragebogen vorfinden.

**Wichtig ist: Die Befragung erfolgt unter Einhaltung aller ethischen und datenschutzrechtlichen Standards rein zu wissenschaftlichen Zwecken. Wir sichern allen Teilnehmerinnen und Teilnehmern zu, dass Ihre Angaben von uns diskret behandelt werden und die Anonymität in der Veröffentlichung garantiert ist.**

Durch Ausfüllen und Abgabe des Fragebogens stimmen Sie folgenden Teilnahmebedingungen zu:

1. Ich erkläre mich einverstanden, dass im Rahmen dieser Befragung anonyme Daten über mich erhoben und in Papierform aufbewahrt sowie auf elektronischen Datenträgern passwortgeschützter Rechner zur wissenschaftlichen Auswertung gespeichert werden.

2. Außerdem erkläre ich mich einverstanden, dass autorisierte und zur Verschwiegenheit verpflichtete zuständige Überwachungsbehörden in die anonymisierten Daten Einsicht nehmen, soweit dies für die Überprüfung der ordnungsgemäßen Durchführung der Studie notwendig ist.

3. Ich bin darüber aufgeklärt, dass ich an der Befragung freiwillig teilnehme und mein Einverständnis erteile. Die Einwilligung zur Erhebung und Verarbeitung meiner anonymen Daten ist unwiderruflich. Aufgrund der anonymen Erhebung der Daten können die gespeicherten Daten rückwirkend nicht mehr gelöscht werden.

4. Ich erkläre mich damit einverstanden, dass meine Daten nach Beendigung der Befragung mindestens zehn Jahre aufbewahrt werden. Danach werden meine Daten gelöscht, soweit nicht gesetzliche oder satzungsmäßige Aufbewahrungsfristen entgegenstehen.

Durch Ausfüllen und Rückgabe des Fragebogens stimmen Sie der Teilnahme an der Befragung zu.

Die Befragung wird in etwa 10-15 Minuten in Anspruch nehmen. Wir würden uns freuen, wenn Sie sich diese Zeit nehmen um den Fragebogen auszufüllen.

**Vielen Dank für Ihre Teilnahme!**

**Studienleitung:**

Titel; Vorname, Name: Prof. Dr. med. Jutta Hübner
Klinik/Institution: Klinik für Innere Medizin II, Hämatologie und internistische Onkologie,

Universitätsklinikum Jena

E-Mail: jutta.huebner@med.uni-jena.de

**Wir beginnen mit ein paar allgemeinen Angaben zu Ihrer Person:**

(Mehrfachauswahl möglich falls notwendig)

| **Geschlecht**: |  weiblich |  männlich |  divers | |  keine Angabe | |
| --- | --- | --- | --- | --- | --- | --- |
| **Alter**: | ______________ | | | | | |
| **Familienstand**: |  ledig |  verheiratet |  geschieden | |  verwitwet |  keine Angabe |
| **Religion**: |  Evangelisch |  Katholisch |  Orthodox | |  Jüdisch |  Muslimisch |
|  | Atheistisch  /keine Religion |  Andere |  keine Angabe | |  |  |
| **Ausbildung**: |  kein Schulabschluss |  Hauptschulabschluss |  Realschulabschluss | |  Abitur/ Fachhochschulreife | |
|  |  Universitätsabschluss |  keine Angabe |  | |  |  |
| **Zeitpunkt der Erstdiagnose:** | Monat: ______________ | | | Jahr: _______________ | | |
| **Krebsart**: |  Brustkrebs   andere gynäkologische Krebsart   Darmkrebs   Krebs des Verdauungstraktes (Magenkrebs, Speisenröhrenkrebs,  Bauchspeicheldrüsenkrebs, Krebs der Gallenwege oder Leber)   Prostatakrebs   Krebs des übrigen Harntraktes (Nieren- oder Harnblasenkrebs)   Lungenkrebs   andere Krebsart, nämlich: _____________________________ | | | | | |
| **Bisherige Behandlungen**: |  Operation |  Chemotherapie |  Bestrahlung | |  Hormontherapie | |
|  |  andere Krebsmedikamente (z.B. Antikörper, zielgerichtete Therapie, Immuntherapie) | |  andere, nämlich: ______________________________ | | | |

**Mit den folgenden Aussagen kommen wir nun zum Hauptteil der Befragung.**

**Nachstehend finden Sie einige Aussagen, die Leute manchmal machen, wenn sie sich über ihre Gesundheit unterhalten. Bitte geben Sie an, wie stark diese Aussagen für Sie zutreffen.**

|  |  | Stimmt nicht (1) | Stimmt kaum (2) | Stimmt eher (3) | Stimmt genau (4) |
| --- | --- | --- | --- | --- | --- |
|  | Letzten Endes bin ich derjenige, der dafür verantwortlich ist, für meine Gesundheit zu sorgen. |  |  |  |  |
|  | Das Wichtigste für meine Gesundheit ist eine aktive Rolle in meiner Gesundheitsversorgung zu übernehmen. |  |  |  |  |
|  | Ich bin überzeugt, dass ich selbst etwas unternehmen kann, um Krankheiten vorzubeugen. |  |  |  |  |
|  | Ich weiss bei jedem meiner Medikamente weshalb ich es nehme. |  |  |  |  |
|  | Ich bin überzeugt, zu wissen, wann ich zum Arzt gehen muss und wann ich ein Gesundheitsproblem selbst behandeln kann. |  |  |  |  |
|  | Ich bin überzeugt, dass ich meinem Hausarzt meine Sorgen mitteilen kann, auch wenn er mich nicht direkt darauf anspricht. |  |  |  |  |
|  | Ich bin überzeugt, dass ich die zu Hause notwendigen medizinischen Behandlungen selbst durchführen kann. |  |  |  |  |
|  | Ich kenne die Ursachen meiner Beschwerden. |  |  |  |  |
|  | Ich kenne verschiedene Behandlungsoptionen für meine Erkrankungen. |  |  |  |  |
|  | Ich war bisher in der Lage, Veränderungen meiner Lebensgewohnheiten − wie gesunde Ernährung und Bewegung – aufrechtzuerhalten. |  |  |  |  |
|  | Ich weiss, wie ich einer Verschlechterung meiner Gesundheit vorbeugen kann. |  |  |  |  |
|  | Ich bin überzeugt, Lösungen zu finden, wenn sich meine Gesundheit verschlechtert. |  |  |  |  |
|  | Ich bin überzeugt, dass ich Veränderungen meiner Lebensgewohnheiten - wie Diät und körperliche Bewegung - auch in stressigen Zeiten fortführen kann. |  |  |  |  |

**Jene Aussagen können mehr oder weniger auf Sie zutreffen.**

**Bitte geben Sie bei jeder Aussage an, inwieweit diese auf Sie persönlich zutrifft und machen Sie ein Kreuz pro Aussage. Bitte lassen Sie keine Antwort aus – bearbeiten Sie alle Fragen.**

|  |  | Trifft überhaupt nicht auf mich zu (0) | (1) | (2) | (3) | Trifft vollständig zu (4) |
| --- | --- | --- | --- | --- | --- | --- |
|  | Das Wichtigste hinsichtlich der Arbeit ist für mich Anständigkeit. |  |  |  |  |  |
|  | Ich übe Kritik an mir, weil ich nicht das tue, was ich versprochen habe zu tun. |  |  |  |  |  |
|  | Mir scheint, dass mir alle Vorwürfe  machen. |  |  |  |  |  |
|  | Ich übe Kritik an mir, weil ich das tue, was ich nicht tun sollte. |  |  |  |  |  |
|  | Wenn ich jemandem etwas Böses getan habe, frage ich mich, was aus ihm wird. |  |  |  |  |  |
|  | Ich bin in Sorge, dass andere Menschen  leiden. |  |  |  |  |  |
|  | Ich habe das Bedürfnis, strenge moralische Regeln zu befolgen. |  |  |  |  |  |
|  | Ich ertrage es nicht, wegen eines Fehlers kritisiert zu werden. |  |  |  |  |  |
|  | Es schmerzt mich, wenn ich andere leiden  sehe. |  |  |  |  |  |
|  | Es gibt für mich nichts wichtigeres als die Pflichten bei der Arbeit und gegenüber anderen Menschen. |  |  |  |  |  |
|  | Ich übe Kritik an mir, weil ich nicht das tue, was ich tun sollte. |  |  |  |  |  |
|  | Ich befürchte immer, für etwas bestraft zu werden. |  |  |  |  |  |
|  | In schwierigen Situationen kann ich mich auf meine Fähigkeiten verlassen. |  |  |  |  |  |
|  | Die meisten Probleme kann ich aus eigener Kraft gut meistern. |  |  |  |  |  |
|  | Auch anstrengende und komplizierte Aufgaben kann ich in der Regel gut lösen. |  |  |  |  |  |

**Abschließend finden Sie hier eine Auflistung einiger Methoden im Rahmen der Komplementär- und Alternativmedizin.**

**Bitte kreuzen Sie an, welche Methoden Sie seit Ihrer Krebsdiagnose nutzen oder genutzt haben und lassen Sie jene Methoden, die Sie nicht nutzen oder nie genutzt haben frei.**

**Wenn Sie keine der genannten Methoden nutzen, kreuzen Sie bitte lediglich das letzte Kästchen an.**

|  |  | *Ja, diese Methode nutze ich / habe ich bereits genutzt* |
| --- | --- | --- |
|  | Vitamin A, C, E oder Betacarotin: |  |
|  | Vitamin B1, B2, B6 oder Folsäure: |  |
|  | Vitamin D: |  |
|  | Vitamin B17 / Aprikosenkerne: |  |
|  | Selen: |  |
|  | Sekundäre Pflanzenstoffe (Curcumin, Lycopin etc.): |  |
|  | Heilpflanzen  *(bitte benennen und*  *den Buchstaben*  *zuordnen):* |   7.1) _______________________________________  7.2) _______________________________________  7.3) _______________________________________  7.4) _______________________________________ |
|  | Chinesische Kräuter/Tees: |  |
|  | Ketogene Diät / Fasten: |  |
|  | Entgiften: |  |
|  | Akupunktur/Akupressur: |  |
|  | Yoga/Tai Chi/Qi Gong: |  |
|  | Sport/Bewegung: |  |
|  | Entspannungsverfahren (Meditation etc.): |  |
|  | Homöopathie, Bachblüten, Schüssler Salze: |  |

| *Ich verwende keine der oben genannten komplementär- und alternativmedizinischen Methoden* |  |
| --- | --- |
